# Probiotics supplement for the prevention of eczema in children: Study protocol for a meta-analysis and systematic review

**DOI:** 10.1097/MD.0000000000016957

**Published:** 2019-08-23

**Authors:** Wenhao Yang, Renyuan Tu, Yanan Hu, Tao He, Weijian Zhang, Li Gu, Hanmin Liu

**Affiliations:** a Department of Pediatrics, West China Second University Hospital, Sichuan University, Chengdu; b Key Laboratory of Birth Defects and Related Diseases of Women and Children (Sichuan University), Ministry of Education, Chengdu; c West China Hospital/West China School of Medicine; d Department of Breast Surgery, West China Hospital/West China School of Medicine, Sichuan University, Chengdu; e Department of Pediatrics, The Affiliated Hospital of Southwest Medical University, Luzhou, Sichuan, China.

**Keywords:** children, eczema, meta-analysis, pregnant woman, probiotics

## Abstract

**Background:**

Atopic dermatitis (AD), also called eczema, is one of the most familiar chronic diseases in childhood. A possible pathological mechanism is immune dysfunction resulting in IgE sensitization to allergens. The recent studies demonstrated that the immune system can be affected by probiotics or prebiotics. However, the effectiveness and safety of probiotics or prebiotics on prevention of eczema are still unclear. To investigate this question, we conduct a systematic review and meta-analysis.

**Methods:**

The protocol followed Preferred Reporting Items for Systematic Reviews and Meta-Analyses Protocols. Four main databases (PubMed, Embase, the Cochrane Library, and the web of science) will be searched dating until 15 July 2019 for randomized controlled trials investigating the effects and safety of probiotics or prebiotics on prevention of eczema in children with no language restrictions. In addition, a manual search of the references of relevant published studies will also be considered.

Studies selection, data extraction, and risk of bias assessment will be conducted by two independent reviewers. The primary outcome is the incidence of eczema. The second outcome is adverse events. The duration of intervention, the timing of intervention and intervention organism will be taken into consideration.

**Results:**

The results will provide useful information about the effect and safety of probiotics or prebiotics on reducing the incidence of eczema in children.

**Conclusion:**

The findings of this study will be published in a peer-reviewed journal.

PROSPERO registration number: CRD42019136528.

## 1 Introduction

Atopic dermatitis (AD), also called eczema, is one of the most familiar chronic diseases in childhood, approximately 10.7% of US children, and related to other clinical features of allergy firmly.^[1,2]^ More than 20% of the population is affected at some time in their life.^[3]^ While the underlying pathogenic mechanisms of eczema are still not drastically understood, there are 2 hypotheses that an immune dysfunction resulting in IgE sensitization to allergens and a defect in the epithelial barrier.^[4,5]^ The recent studies demonstrated that the immune system can be affected by probiotics or prebiotics.^[6]^

Probiotics are a group of active microorganisms that are beneficial to the host.^[7]^ The expansion and differentiation of Treg cells are also promoted by the probiotic community and a TGF-β-rich environment created by probiotics.^[6]^ Through fermenting fibers, probiotics stimulate the production of metabolites such as short-chain fatty acids, inhibiting histone deacetylase activity, the majority of which are acetate, propionate, and butyrate.^[8,9]^ Local DCs which migrate to the draining lymph nodes can be activated by bacterial metabolites or bacteria themselves to activate naive T cells to effector T cells, Tregs, or Th17 cells, which can migrate back to the gut mucosa or enter the systemic circulation.^[10]^ The World Allergy Organization (WAO) also took it into consideration that there is a likely benefit from using probiotics for preventing eczema.^[11]^ However, the recommendations are very conditional due to the very low quality of evidence.

Recently, there are many controversial results from several RCTs evaluating the effect of probiotics or prebiotics on reducing the incidence of eczema.^[12–14]^ It's unclear about what is a suitable type of pro/prebiotic, what is the effective dose, and when to start or stop. Therefore we conducted this meta-analysis to explore whether probiotics supplementation during pregnancy and infancy can prevent eczema in children.

## 2 Methods

### 2.1 Registration

Our study protocol has been registered in the PROSPERO and the registration number is CRD42019136528. This meta-analysis and systematic review will follow the guideline of the *Cochrane Handbook for Systematic Reviews of Interventions*^[15]^ and the PRISMA (Preferred Reporting Items for Systematic Reviews and Meta-Analyses) statement^[16]^ and the software RevMan 5.3 and STATA version 14.0 (College Station, TX) will be used to construct the meta-analysis. This study does not require ethical approval because there is no direct involvement of human.

### 2.2 Eligibility criteria

The eligibility criteria are summarized using the PICOS approach (patients, intervention, comparisons, outcomes and study design type).

### 2.3 Types of participants

Eligible participants include healthy pregnant women and newborns who have a family history of atopic disease; that is, >1 family member (mother, father, or older sibling) with allergic diseases, allergic rhinitis, or asthma and a confirmed allergic sensitization against an inhalant allergen. There are no restrictions on age, ethnic distribution, and gender.

### 2.4 Interventions and comparisons

The treatment group will receive pro/prebiotics therapy. The control group will receive placebo for the same time. Those trials reported their participants to receive other probiotics or antibiotics will be excluded.

### 2.5 Outcome measures

Incidence of eczema is the primary outcome. The second outcome is adverse events. We will also focus on the duration of intervention, the timing of intervention, intervention organism, follow-up time and different regions.

### 2.6 Types of studies

Randomized controlled trials (RCTs) published with no language restriction dating until 15 July 2019 will be included.

### 2.7 Search methods

PubMed, EMBASE, the Cochrane Library and web of science will be systematically searched for eligible studies dating until 15 July 2019. The terms including eczema, probiotic, prebiotic, pregnant, child and RCT will be involved in the search strategy. A detailed search strategy in PubMed, EMBASE, the Cochrane Library and web of science is described in Table 1. Relevant studies and systematic reviews will also be scanned for additional eligible trials.

**Table 1:**
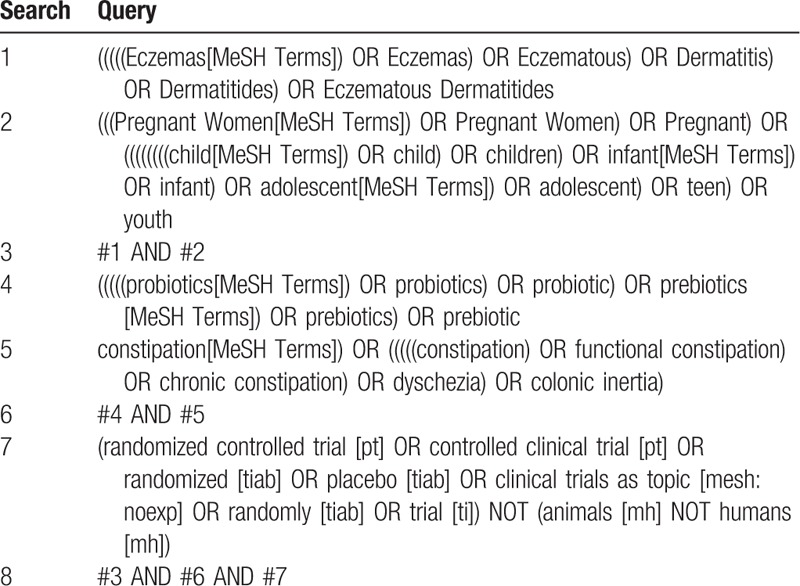
Preliminary search strategy in PubMed.

## 3 Study selection and data extraction

### 3.1 Study selection

**Figure 1:**
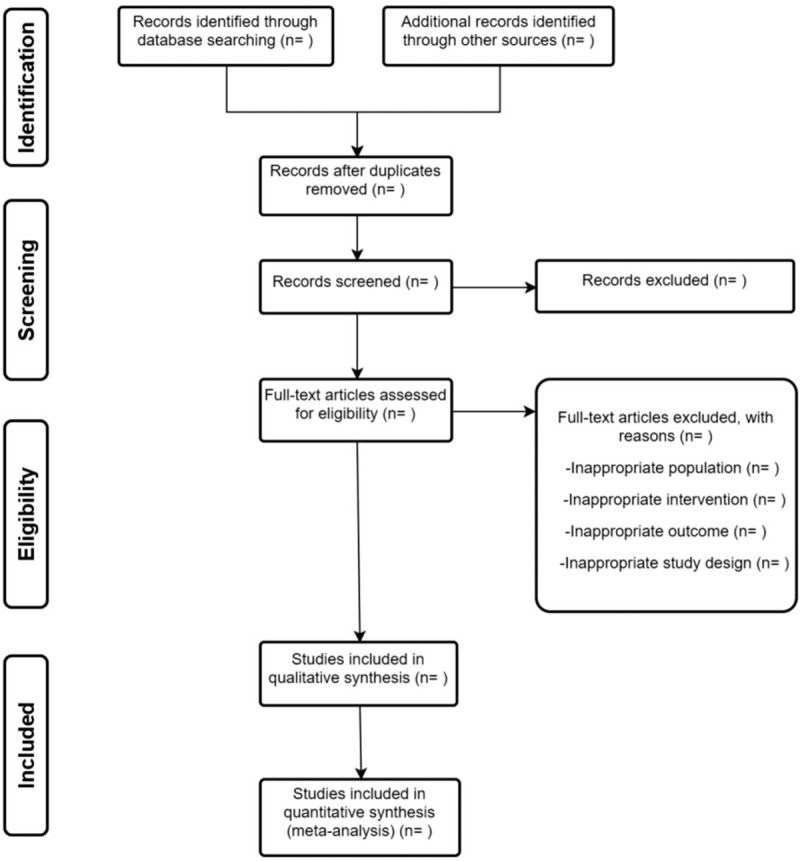
Flow diagram of study selection.

Study selection will be performed by two reviewers independently. The search results from four electronic databases and additional trials from other relevant articles s will be sent to Endnote. After duplicates removed, most of the trials will be excluded by scanning the title and abstract. Secondly, full texts will be read for further exclusion. The selection process will be summarized in a PRISMA flow diagram (Fig. 1). Any disagreements will be resolved with the help of a third author.

### 3.2 Data extraction

Two reviewers will extract relevant data independently from the included studies. Study characteristics, patient characteristics, data needed for quality assessment, and outcomes will be included. Participant characteristics include the type of interventions received, mean age, sex, sample and family history. Outcomes include the incidence of eczema and adverse events. The duration of intervention, the timing of intervention, intervention organism, follow-up time and participant regions will also be extracted. All study characteristics will be collated in the same standardized collection form. Two reviewers will check the data with each other when extraction finished. Any discrepancies should be resolved by negotiation between the 2 reviewers with the help of a third author.

### 3.3 Risk of bias assessment

The methodological quality of all included studies will be independently assessed by two authors based on the Cochrane Collaboration's tool.^[17]^ The following contents will be evaluated: random sequence generation, allocation concealment, blinding of participants and personnel, blinding of outcome assessment, incomplete outcome data, selective reporting, and other biases. Each domain will be judged by the level of risk of bias: high level, low level or unclear level. Any disagreements will be solved by discussion.

## 4 Data synthesis and statistical analysis

### 4.1 Data synthesis

The RevMan 5.3 software and STATA version 14.0 (College Station, TX) will be used to construct the meta-analysis. Dichotomous data, the incidence of eczema, will be reported as risk ratios (RRs) with their 95% confidence intervals (CIs). The mean difference (MD) and the 95% confidence interval (CI) will be calculated for the continuous variable. *P* < .05 will be considered to be statistically significant.

### 4.2 Assessment of heterogeneity

Heterogeneity will be assessed by the *χ*^2^ test and the *I*^2^ test. If P > .10 and *I*^2^ < 50%, the heterogeneity is acceptable and a fixed-effect model will be used for data analysis. If *P* < .10 and I^2^ ≥ 50%, we will search for the reasons for the high heterogeneity and use a random-effects model for data analysis.

### 4.3 Sensitivity analysis

Sensitivity analysis will be carried out based on the sample size, the missing data result and the methodological quality of the included study. If necessary, we will exclude a low-quality study and repeat the meta-analysis to test the stability of the pooled results

### 4.4 Assessment of reporting bias

If more than 10 studies are included, the reporting bias will be assessed by a Begg funnel plot and Egger regression. The results will be calcified based on the Cochrane Handbook for Systematic Reviews of Interventions.

### 4.5 Confidence in cumulative evidence

The quality of evidence will be assessed based on the Grading of Recommendations Assessment, Development, and Evaluation (GRADE) system. The evidence will be adjusted to 4 levels: high, moderate, low, or very low.

## 5 Discussion

Currently, whether to use pro/prebiotics in pregnant woman or children with high-risk eczema remains a controversy. Some published systematic review and meta-analysis shows the different effects of various probiotics in pregnant woman or children. It's hard to judge which probiotics play a big role in reducing the incidence of eczema. What is the dose of effective probiotic or prebiotic? What is the best time of the administration of probiotics or prebiotic? How to choose a suitable type of probiotics or prebiotic? These practical problems remain unclear because of the absence of evidence. Therefore, it is very necessary to conduct a systematic review and meta-analysis to investigate the effects and safety of probiotic or prebiotic for reducing the incidence of eczema in high-risk pregnant woman or children. We aim to summarize direct evidence about published RCTs and provide evidence-based suggestions for the clinical use of probiotic or prebiotic.

## Author contributions

WHY put forward the concept of this study. WHY drafted the preliminary version of this protocol. TH and RYT will contribute to the study search, study selection, data extraction, and risk of bias assessment. WJZ, YN Hu and LG will complete the data analysis. WHY, LG and HML will help to solve any disagreement and ensure the quality of this study. All authors critically reviewed, revised and approved the final manuscript.

**Conceptualization:** Wenhao Yang, Weijian Zhang

**Data curation:** Wenhao Yang, Tao He, Renyuan Tu.

**Methodology:** Weijian Zhang, Li Gu, Yanan Hu.

**Project administration:** Wenhao Yang.

**Supervision:** Hanmin Liu.

**Writing – original draft:** Wenhao Yang.

**Writing – review & editing:** Wenhao Yang, Li Gu, Hanmin Liu.
